# The Increased Expression of Integrin α6 (ITGA6) Enhances Drug Resistance in EVI1^high^ Leukemia

**DOI:** 10.1371/journal.pone.0030706

**Published:** 2012-01-25

**Authors:** Norio Yamakawa, Kazuko Kaneda, Yusuke Saito, Emi Ichihara, Kazuhiro Morishita

**Affiliations:** Division of Tumor and Cellular Biochemistry, Department of Medical Sciences, Faculty of Medicine, University of Miyazaki, Miyazaki, Japan; National Cancer Center, Japan

## Abstract

Ecotropic viral integration site-1 (EVI1) is one of the candidate oncogenes for human acute myeloid leukemia (AML) with chromosomal alterations at 3q26. High EVI1 expression (EVI1^high^) is a risk factor for AML with poor outcome. Using DNA microarray analysis, we previously identified that integrin α6 (ITGA6) was upregulated over 10-fold in EVI1^high^ leukemia cells. In this study, we determined whether the increased expression of ITGA6 is associated with drug-resistance and increased cell adhesion, resulting in poor prognosis. To this end, we first confirmed the expression pattern of a series of integrin genes using semi-quantitative PCR and fluorescence-activated cell sorter (FACS) analysis and determined the cell adhesion ability in EVI1^high^ leukemia cells. We found that the adhesion ability of EVI1^high^ leukemia cells to laminin increased with the increased expression of ITGA6 and integrin β4 (ITGB4). The introduction of small-hairpin RNA against EVI1 (shEVI1) into EVI1^high^ leukemia cells reduced the cell adhesion ability and downregulated the expression of ITGA6 and ITGB4. In addition, the overexpression of EVI1 in EVI1^low^ leukemia cells enhanced their cell adhesion ability and increased the expression of ITGA6 and ITGB4. In a subsequent experiment, the introduction of shRNA against ITGA6 or ITGB4 into EVI1^high^ AML cells downregulated their cell adhesion ability; however, the EVI1^high^ AML cells transfected with shRNA against ITGA6 could not be maintained in culture. Moreover, treating EVI1^high^ leukemia cells with neutralizing antibodies against ITGA6 or ITGB4 resulted in an enhanced responsiveness to anti-cancer drugs and a reduction of their cell adhesion ability. The expression of ITGA6 is significantly elevated in cells from relapsed and EVI1^high^ AML cases; therefore, ITGA6 might represent an important therapeutic target for both refractory and EVI1^high^ AML.

## Introduction

Ecotropic viral integration site-1 (EVI1) is an oncogenic transcription factor for murine and human myeloid leukemia [1], [2]. Human EVI1 is localized on chromosome 3q26 [3]. Although only approximately 1 to 3% of acute myeloid leukemia (AML) cases result from a translocation in 3q26, the elevated expression of EVI1 has been detected in 5% to 10% of AML cases in the absence of chromosomal abnormalities at 3q26 [4]. AML with EVI1 high expression (EVI1^high^) is a poor prognosis subtype of AML that does not respond to currently available treatments [5].

EVI1 is a nuclear transcription factor with a DNA-binding zinc finger, an acidic amino acid cluster region and C-terminal binding protein (CtBP) motifs [6], [7]. Although EVI1 has been reported to transcriptionally repress or suppress TGFb signaling by recruiting Smad3 and the co-repressor CtBP [8]–[10], we showed that EVI1 is directly associated with the GATA-2 promoter and upregulates GATA-2 transcription to maintain hematopoietic stem cells (HSCs) and AML with EVI1^high^ expression [11], [12] in EVI1-deficient mice. In addition to the observed reduction in GATA-2 expression, other important factors for HSC maintenance, including Angiopoietin-1 and Tie-2, were also de-regulated in EVI1-deficient mice [11]. These results suggest that murine Evi1 might de-regulate transcription factors or other signal transduction molecules necessary for HSC maintenance [11]. However, we do not know precisely how Evi1 is involved in the maintenance of HSCs.

Recently, there has been increased interest in understanding the regulatory interactions between osteoblasts and HSCs in the bone marrow microenvironment. Individual HSCs are typically anchored to the stroma via a network of adhesion molecules [13], [14]. Recent studies have indicated the importance of these adhesion molecules (integrins and cadherins) in hematopoietic stem cell development and have shown that they function as key elements for the detection and translation of the extrinsic cues provided by the hematopoietic microenvironmental niche [15], [16]. The integrins are heterodimeric complexes composed of two noncovalently associated transmembrane glycoprotein subunits: one from sixteen different alpha (a) subunits and the other from eight different beta (b) subunits [17], [18]. abVery late antigen 4 (VLA4), a a4/b1 integrin heterodimer, participates in both cell-cell and cell-matrix interactions with vascular cell adhesion molecule-1 (VCAM1) and fibronectin (FN). In adult mice, VLA4/VCAM1 interactions are key elements in the mobilization and homing of hematopoietic stem cells to bone marrow [19]. Moreover, treatment with anti-VLA4 antibodies mobilizes CD34+ hematopoietic progenitor cells from the bone marrow to the peripheral blood [20]. Studies addressing the role of VLA4 in AML cell lines have described the drug resistance induced by the interaction of tumor cells with stromal cells or the extracellular matrix (ECM) as cell adhesion-mediated drug resistance (CAM-DR) [21], [22]. In an analysis of 175 adult AML patients, however, VLA4 expression was not significantly associated with the response to anti-cancer drugs or with relapse-free or overall survival rates [23]. Thus, other adhesion molecules may also be important in the maintenance of HSC and leukemia stem cells.

In this study, we observed that the cell adhesion ability of the EVI1^high^ AML cells was higher than that of the EVI1^low^ cells. The increased adhesion of EVI1^high^ AML cells was dependent on the expression of the integrins α6 (ITGA6) and β4 (ITGB4) in complex with laminin, particularly laminin-332. Because the increased adhesion ability of the AML cells increased their resistance to chemotherapy, and the expression of ITGA6 was significantly higher in AML cases in relapse, ITGA6 might be a novel molecular target in EVI1^high^ leukemia cells.

## Results

### Overexpression of ITGA6 enhances the ability of EVI1^high^ leukemia cell lines to adhere to matrigel and laminin

**Figure 1:**
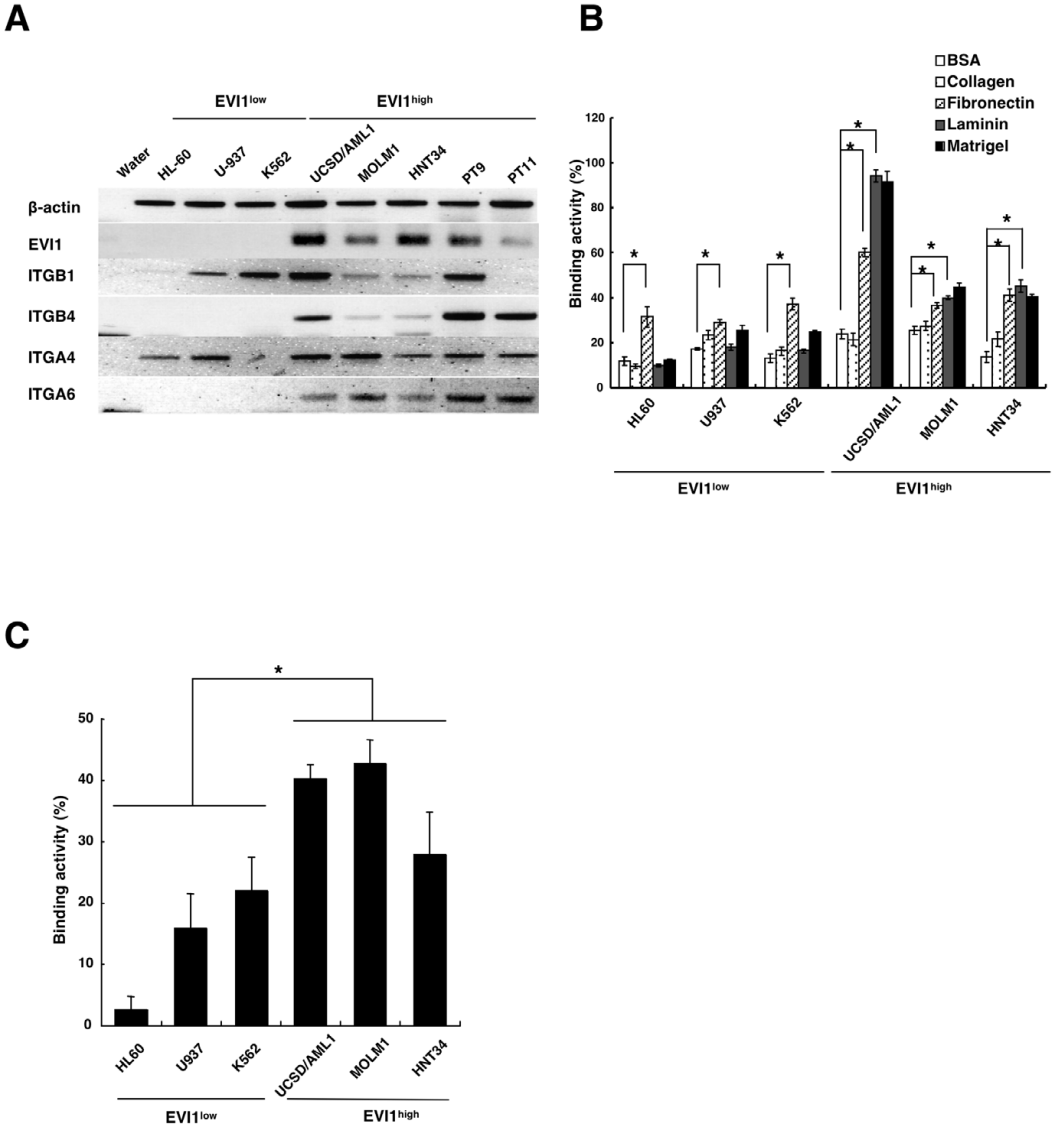
Higher cell adhesion ability in AML cell lines with EVI1^high^ expression. **A**. The expression of four integrin genes (ITGB1, ITGB4, ITGA4 and ITGA6), EVI1 and b-actin as a control was determined by semiquantitative RT-PCR in three different EVI1^low^ and EVI1^high^ AML cell lines and two primary AML cells lines with high EVI1 expression (PT9 and PT11). **B**. Six AML cell lines with low or high EVI1expression, as indicated in the figure, were incubated in culture medium on BSA, collagen, fibronectin, laminin or matrigel-coated plates; the percentage of the total number of incubated cells that adhered to the plates was designated as the binding activity (%). Each experiment was performed in triplicate, and the experiments were independently repeated at least three times. The data are given the as the mean ± standard error (S.E). The statistical analysis was performed using the Student's *t-test* (*p<0.05, vs. BSA-coated plate). **C**. Six AML cell lines were incubated with the murine osteoblastic cell line MC3T3-E1, and the percentage of cells that bound to MC3T3-E1 cells was determined. Each experiment was performed in triplicate, and the experiments were independently repeated at least three times. The statistical analysis was performed using the Student's *t-test* (*p<0.05, vs. EVI1^low^ cell lines).

To search for novel molecular targets in refractory myeloid leukemia with high EVI1 expression, we previously analyzed the gene expression profiles of 12 human myeloid cell lines using an oligonucleotide microarray (Human Genome U133 Plus 2.0 Array; Affymetrix) containing 38,500 genes [24]. We identified ITGA6 in 26 selected genes that were upregulated over ten-fold in EVI1^high^ leukemia cells (*p*<0.01). To confirm the results of the microarray analysis, the expression levels of a series of integrin genes were determined in three EVI1^low^ and EVI1^high^ cell lines using semi-quantitative RT-PCR. We determined the levels of the eight different β integrin genes (1 through 8), fifteen different α integrin genes (1 through 10, E, L, M, V, and X) and two cadherin molecules (N and VE). As shown in **Fig. S1A**, ITGB1, ITGB3, ITGA4, and ITGA5 were expressed in five of the six leukemia cell lines, and ITGB4 and ITGA6 were specifically expressed in the three EVI1^high^ leukemia cell lines. To confirm the results of the expression patterns of the integrin genes, we used the six cell lines and two primary leukemia cell lines from patients with the inv(3) (q21q26) subtype (PT9 and PT11) [24] to determine the expression of ITGB1, ITGB4, ITGA4, and ITGA6. ITGB1 and ITGA4 were expressed as very late antigen-4 (VLA-4) in most of the cell lines, but ITGB4 and ITGA6 were expressed in all three leukemia cell lines and in the two primary EVI1^high^ leukemia cell lines (**Fig. 1A**).

Because a number of integrin genes are involved in binding to fibronectin, laminin and matrigel [15], [16], we compared the cell adhesion ability of EVI1^high^ and EVI1^low^ leukemia cell lines. The three human EVI1^low^ myeloid cell lines (HL60, U937, and K562) showed a reduced binding affinity to laminin and matrigel but an increased binding affinity to fibronectin. In contrast, the three EVI1^high^ cell lines (UCSD/AML1, MOLM1, and HNT34) exhibited an increased affinity for laminin, matrigel and fibronectin, with significant differences (**Fig. 1B**). Because the primary component of the matrigel used in this experiment is the laminin complex, the enhanced cell adhesion ability of EVI1^high^ leukemia cells might depend on binding to the laminin complex.

We also investigated the ability of various leukemia cells to adhere to the murine bone marrow stromal/osteoblastic cell line MC3T3-E1. As shown in Fig. 1D, a higher percentage of cells in the three EVI1^high^ leukemia cell lines (UCSD/AML1, MOLM1 and HNT34) were attached to the MC3T3-E1 cells compared with the three EVI1^low^ leukemia cell lines (HL60, K562 and U937), and this difference was statistically significant (p<0.01). Because the EVI1^high^ leukemia cell lines exhibited an enhanced ability to adhere to the laminin complex (**Fig. 1C**), we propose that the laminin complex might be one of the main molecular targets of this increased adhesion to the MC3T3-E1 cells.

### The expression of ITGA6 and ITGB4 is dependent on the expression of EVI1

**Figure 2:**
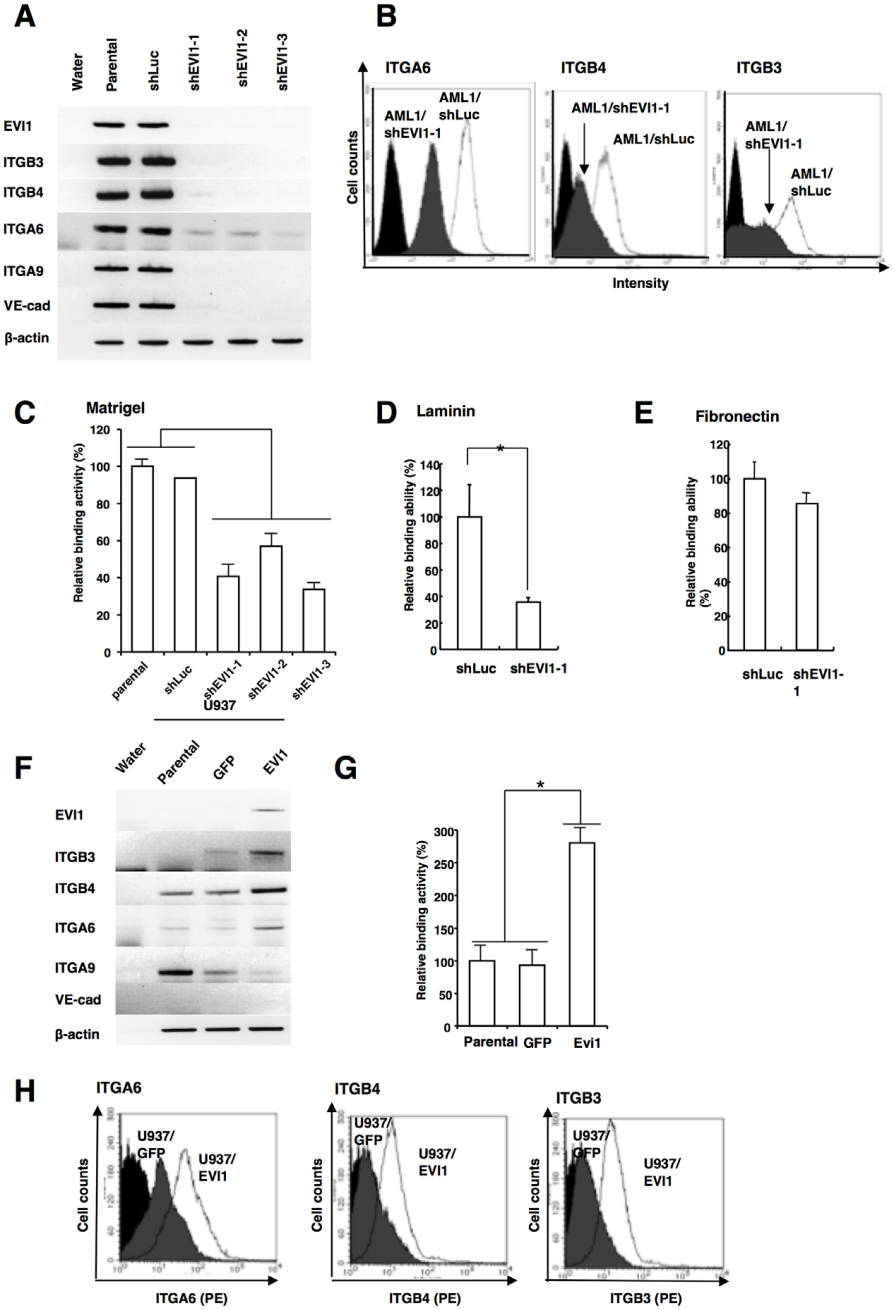
The expression of ITGA6 and ITGB4 is specifically dependent on the expression of EVI1. **A**. The pattern of expression of EVI1 and five integrin genes (ITGB3, ITGB4, ITGA6, ITGA9 and VE-cadherin) in various UCSD/AML1 cell lines. Three cell lines with small hairpin RNA (shRNA) against EVI1 (shEVI1-1, -2 and -3) and the parental and UCSD/AML1 cell lines harboring an expression vector for shRNA against firefly luciferase were used to determine the levels of expression of five integrin (ITGB3, ITGB4, ITGA6, ITGA9 and VE-cadherin) and control (EVI1 and b-actin) genes using RT-PCR. **B**. The expression of ITGA6, ITGB4 and ITGB3 in AML1/shLuc and AML1/shEVI1-1 was determined using FACS analysis after staining the cells with PE-conjugated antibodies to specific integrins. **C**. The same cell lines described in (2a) were assessed for their ability to adhere to the matrigel. The relative binding activity was calculated by comparison to the basal binding activity of the parental UCSD/AML1 cell line. **D and E**. The binding activity of AML1/shLuc and AML1/EVI1-1 cells to laminin (D) and fibronectin (E) was compared. Both cell lines were cultured on laminin or fibronectin-coated plates. **F and G**. After the EVI1 expression vector was introduced into the U937 cell line, the expression of five integrin genes (ITGB3, ITGB4, ITGA6, ITGA9, VE-cadherin) and EVI1 was determined using RT-PCR. b-actin was used as a control (F). The cell adhesion ability of the U937/EVI1, U937/parental and U937/GFP cell lines was determined (G). **H**. The expression of ITGA6, ITGB4 and ITGB3 in U937/GFP and U937/EVI1 was determined using FACS analysis after staining the cells with PE-conjugated antibodies to specific integrins. Each experiment shown in Figure **2C, 2D, 2E, and 2G** was performed in triplicate, and the experiments were independently repeated at least three times. The data are given as the mean ± S.E. The statistical analysis was performed using the Student's *t-test* (**p<0.05, vs. each control).

To determine which of the integrin genes were dependent on EVI1 expression, small hairpin RNA against EVI1 (shEVI1) were introduced into UCSD/AML1 cells with EVI1^high^. Three UCSD/AML1 cell lines expressing shEVI1 (AML1/shEVI1-1 to -3), and a control cell line expressing an shRNA for firefly luciferase (shLuc) (AML1/shLuc) were established. Because the EVI1 expression was significantly downregulated in the AML1/shEVI1-1 to 3 cell lines (**Fig. 2A**), we determined the expression of a series of integrin genes using semi-quantitative RT-PCR (**Fig. S1B and** **Fig. 2A**). The results showed that the expression of ITGB3, ITGB4, ITGA6, ITGA9, and VE-cadherin was downregulated along with EVI1 in the AML1/shEVI1-1 to 3 cell lines. The high expression of ITGB3, ITGB4 and ITGA6 in the UCSD/AML1 cells and the downregulation of ITGA6, ITGB4 and ITGB3 in the AML1/shEVI1-1 cells were confirmed through immunofluorescence staining and FACS analysis (**Fig. 2B**). Moreover, ITGB4, ITGA6, ITGA9, and VE-cadherin were also downregulated in the HNT34 cell line, and PT9 and PT11 primary AML cell lines upon shEVI1 transfection, which was associated with a reduction in matrigel cell adhesion (**Figs. S1C to F**). Taken together, the results demonstrated that ITGB4, ITGA6, ITGA9, and VE-cadherin are the candidate integrin genes with increased adhesion ability in EVI1^high^ leukemia cells. Moreover, we determined the cell adhesion ability of the AML1/shEVI1 cell lines and found that the adhesion of AML1/shEVI1-1 to 3 cells to matrigel was significantly reduced compared with that of the parental UCSD/AML1 and AML1/shLuc cells (**Fig. 2C**). The cell adhesion of the AML1/shEVI1-1 cells to laminin, but not to fibronectin, was also significantly reduced compared with that of the AML1/shLuc cells (**Fig. 2D and E**).

**Figure 3:**
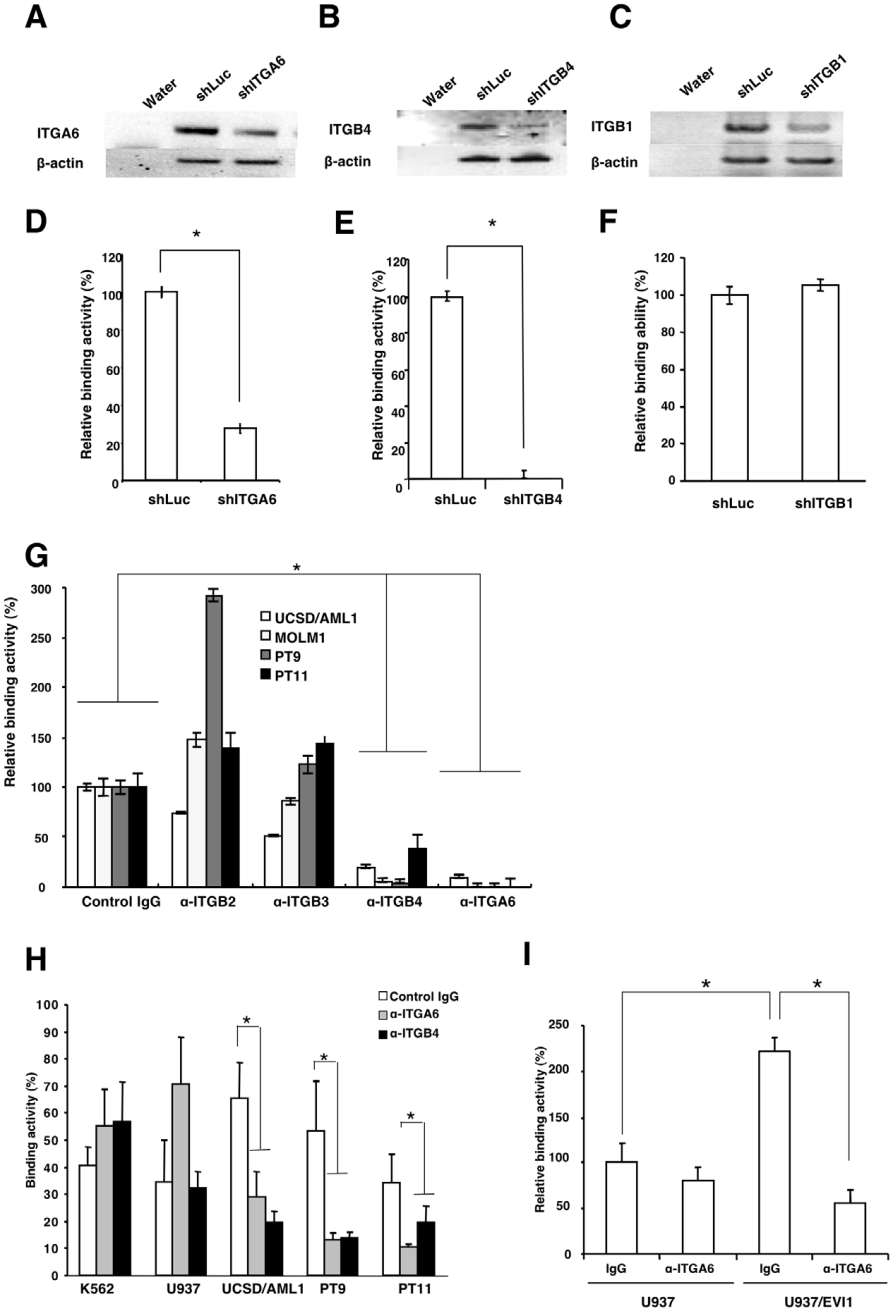
The adhesion ability of EVI1^high^ leukemia is specifically dependent on the expression of ITGA6 and ITGB4. **A, B, and C**. UCSD/AML1 cells were transiently transfected with expression vectors for shRNA against ITGA6 (shITGA6), ITGB4 (shITGB4), or ITGB1 (shITGB1), and the expression of ITGA6, ITGB4 and ITGB1 was determined using RT-PCR. UCSD/AML1 cells transfected with shRNA for firefly luciferase (shLuc) were used as a control, and the expression of b-actin was used as an internal control. **D, E, and F**. The cell adhesion ability of the cells in A, B, and C was determined through co-culture with the MC3T3-E1 cell line. The relative binding activity was calculated by comparison to the basal binding activity of control AML1/shLuc cells. **G**. The effect of neutralizing antibodies to the integrins on the cell binding activity of EVI1^high^ leukemia cells. Two leukemia (UCSD/AML1 and MOLM1) and two primary human AML (PT9 and PT11) cell lines were cultured on matrigel-coated plates in the presence or absence of anti-ITGA6, ITGB2, ITGB3, or ITGB4 antibodies. The relative binding activity was calculated by comparison to the basal binding activity of each cell line treated with the control isotype IgG. **H**. Two EVI1^low^ (K562 and U937) and three EVI1^high^ (UCSD/AML1, PT9 and PT11) leukemia cell lines were treated with anti-ITGA6 or ITGB4 antibodies or with the control IgG, and the binding of each cell line to the matrigel-coated plates was determined. **I**. The parental and EVI1-expressing (U937/EVI1) U937 cell lines were treated with anti-ITGA6 or control IgG, and their binding to the MC3T3-E1 cell line was determined. The relative binding activity was calculated by comparison to the basal binding activity of the parental U937 cells treated with the control isotype IgG. Each experiment was performed in triplicate, and the experiments were independently repeated at least three times. The data are given as the mean ± S.E. The statistical analysis was performed using the Student's *t-test* (*p<0.05, vs. each control).

In a subsequent experiment, we transiently introduced EVI1 and GFP expression vectors into the EVI1^low^ U937 cells and determined the resulting levels of expression of the various integrin genes. The adhesion of U937/EVI1 cells to matrigel was increased three-fold, and the expression of ITGB3, ITGB4 and ITGA6 was also increased in U937/EVI1 cells relative to their adhesion ability and expression in the U937/parental and U937/GFP cells (**Fig. 2F** **and Fig. S1G**). Because ITGB4 and ITGA6 form a heterodimer that binds to the laminin receptor and treatment with a neutralizing antibody to ITGB3 did not suppress the cell adhesion ability of EVI1^high^ leukemia cells (Fig. 3G), we further characterized the relationship between the expression of ITGA6/ITGB4 and the increased adhesion ability of the EVI1^high^ leukemia cells.

### The adhesion ability of leukemia cells with EVI1^high^ expression is specifically dependent on the expression of ITGA6 and ITGB4

To evaluate whether the adhesion ability of the EVI1^high^ leukemia cells was specifically dependent on the expression of ITGA6 or ITGB4, shRNA against ITGA6 or ITGB4 was introduced into UCSD/AML1 cells using an Amaxa Nucleofector, and the cell adhesion ability to matrigel was determined. The expression of ITGA6 and the cell adhesion ability were significantly reduced in UCSD/AML1 cells transfected with shITGA6 (AML1/shITGA6) (**Fig. 3A and D**). Similar results were obtained in UCSD/AML1 cells transfected with shITGB4 (AML1/shITGB4) (**Fig. 3B and E**). The growth rate of the AML1/shITGA6 cells was reduced, and these cells could not be maintained in culture, whereas the AML1/shITGB4 grew at rate similar to the parental UCSD/AML1 control cells, suggesting that the signal transduction of ITGA6 is essential for the maintenance of leukemia cells. Because ITGA6 forms a heterodimer with ITGB4 or ITGB1, shITGB1 was introduced into UCSD/AML1 cells, and their adhesion to matrigel was measured. The adhesion of UCSD/AML1 cells to matrigel did not change significantly upon transfection with shITGB1 (**Fig. 3C and F**). These results suggest that the ITGA6 and ITGB4 heterocomplex is the primary cell adhesion molecule on EVI1^high^ leukemia cells.

To confirm the ITGA6 dependence of the increased cell adhesion of EVI1^high^ leukemia cells, we used a series of neutralizing antibodies. Two leukemia (UCSD/AML1 and MOLM1) and two primary human AML (PT9 and PT11) cells lines were cultured on matrigel and treated with or without anti-ITGA6, ITGB2, ITGB3, or ITGB4 antibodies. Two days after treatment, the binding activity of all four cell lines was significantly reduced by the treatment with anti-ITGA6 or anti-ITGB4 antibodies; however, isotype IgG, anti-ITGB2 or anti-ITGB3 antibodies did not inhibit the binding of EVI1^high^ leukemia cells to matrigel (**Fig. 3G**).

Because the EVI1^high^ leukemia cells exhibited an increased adherence to the murine osteoblastic cell line MC3T3-E1 (**Fig. 1D**) we examined whether this adhesion was dependent on ITGA6 or ITGB4 expression by treating with a neutralizing antibody. Two EVI1^low^ (K562 and U937) and three EVI1^high^ (UCSD/AML1, PT9 and PT11) leukemia cell lines were co-cultured with MC3T3-E1 cells, and the mixed cultures were subsequently treated with anti-ITGA6 or anti-ITGB4 antibody or the control isotype anti-rabbit IgG. The ability of the three EVI1^high^ leukemia cells to bind to MC3T3-E1 cells was significantly reduced upon treatment with anti-ITGA6 or anti-ITGB4 antibody, whereas the binding ability of the two EVI1^low^ leukemia cells did not change (**Fig. 3H**). To confirm this result, we examined the adhesion of U937/parental and U937/EVI1 cells to matrigel upon treatment with anti-ITGA6 antibody. The anti-ITGA6 antibody significantly inhibited the cellular adhesion of U937/EVI1 cells to matrigel, but did not inhibit the cell adhesion of the parental U937 cells (**Fig. 3I**). Therefore, the expression of ITGA6 and ITGB4 was EVI1 dependent, and the increased expression of ITGA6 and ITGB4 in EVI1^high^ leukemia cells increased their ability to adhere to the osteoblastic MC3T3-E1 cell line.

### The downregulation of laminin-332 in osteoblastic MC3T3-E1 cells partially inhibits binding to EVI1^high^ leukemia cells

**Figure 4:**
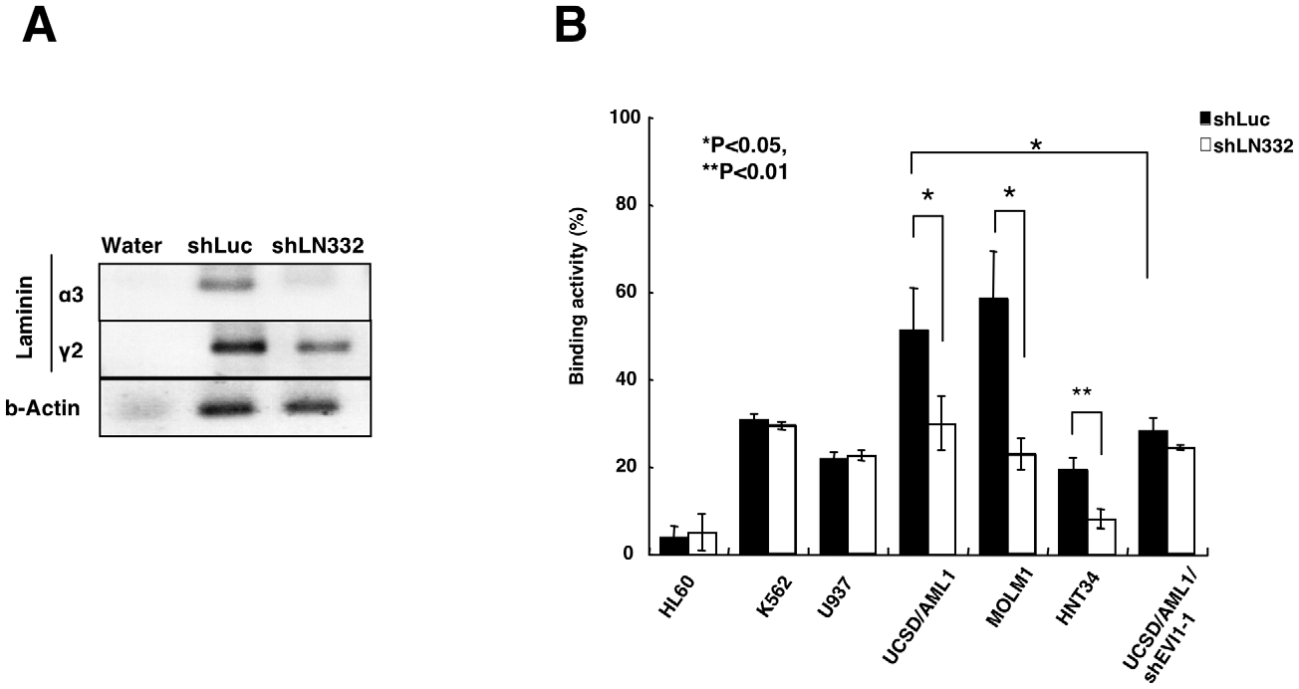
Laminin-332 on MC3T3-E1 cells is a main target for binding to EVI1^high^ leukemia cells. **A**. The expression of laminin a3 and g2 in MC3T3-E1 cells transfected with shRNA for firefly luciferase (shLuc) or laminin a3 (shLN332) was determined using RT-PCR. The expression of b-actin was used as an internal expression control. **B**. The binding of various myeloid leukemia cell lines to the two cell lines MC3T3-E1/shLuc or/shLN332 was determined. Each experiment was performed in triplicate, and the experiments were independently repeated at least three times. The data are given as the mean ± S.E. The statistical analysis was performed using the Student's *t-test* (**p<0.01; *p<0.05, vs. control).

The ITGA6/IGTB4 heterocomplex has a clear specificity for laminin-332 (a3b3g2) or laminin-511 (a5b1g1) [25]. Therefore, we measured the expression of a series of laminin a, b, and g chains in MC3T3-E1 cells; the expression of each laminin chain was clearly detected in these cells (**Fig. S2**). We subsequently introduced shRNA against the a3 chain of laminin-332 (shLN332) into MC3T3-E1 cells and measured the ability of the MC3T3-E1 cells to adhere to various types of myeloid leukemia cells. After the introduction of shLN332 or shLuc into MC3T3-E1 cells (MC3T3-E1/shLN332 or MC3T3-E1/shLuc, respectively), the expression of the a3 chain of laminin-332 was significantly reduced (**Fig. 4A**), but the expression of the g3 chain of laminin-332 and b-actin were not changed. Compared to the MC3T3-E1/shLuc cells, the MC3T3-E1/shLN332 cells displayed significantly reduced adhesion to the EVI1^high^ cell lines UCSD/AML1, MOLM1 and HNT34. However, their adhesion to the leukemia cell lines with EVI1^low^ expression, such as HL60, K562, and U937, did not change (**Fig. 4B**). Moreover, the UCSD/AML1/shEVI1 cells displayed reduced adhesion to the MC3T3-E1/shLuc cells compared with that of the UCSD/AML1 cells, whereas the adhesion of the UCSD/AML1/shEVI1 cells to the MC3T3-E1/shLuc and MC3T3-E1/shLN332 cells did not change. These data suggest that the increased adhesion of the leukemia cell lines with EVI1^high^ expression is mainly dependent on the laminin complex, particularly laminin-332.

### EVI1^high^ leukemia cells treated with ITGA6 or ITGB4- neutralizing antibodies or small hairpin RNA against EVI1 or ITGB4 recover drug sensitivity

**Figure 5:**
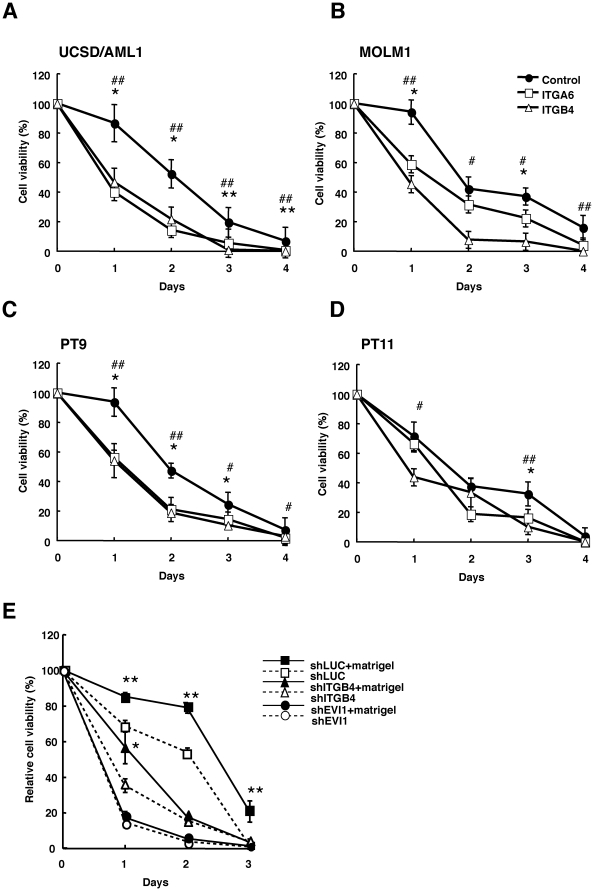
Drug sensitivity of EVI1^high^ leukemia cells is restored by treatment with neutralizing antibodies to ITGA6 or ITGB4 or by transfection with small hairpin RNAs against EVI1 or ITGB4. **A–D**. Two myeloid leukemia (UCSD/AML1 and MOLM1) and two primary AML (PT9 and PT11) cell lines were treated with 1×10^−6^ M Ara-C and anti-ITGA6 or ITGB4 antibodies for four days, and the viable cells were counted at each indicated time point. The percent cell viability in comparison with the number of untreated cells on each indicated day is shown. **E**. The UCSD/AML1 cells expressing shRNA for firefly luciferase (shLuc), ITGB4 (shITGB4) or EVI1 (shEVI1) were treated with Ara-C on BSA or matrigel-coated plates for three days. The viable cells were counted at each time point. The relative cell viability is expressed as a percentage of the viability of the untreated cells. Each experiment was performed in triplicate, and the experiments were independently repeated at least three times. The results are given as the mean ± S.E. The statistical analysis was performed using the Student's *t-test* (**p<0.05, vs. control).

We next determined whether treating EVI1^high^ leukemia cells with a neutralizing antibody against integrin improves the drug sensitivity of the cells in the adhered state. Two EVI1^high^ leukemia cell lines, UCSC/AML1 and MOLM1, and two primary leukemia cell lines, PT9 and PT11, were used in this experiment; the IC50 of Ara-C for these cells cultured on the matrigel coated plates was approximately 10^−6^ M (**Fig. S3**). The four cell lines were seeded on matrigel-coated culture plates ith or without anti-ITGA6 or anti-ITGB4 antibodies in the presence of 10^−6^ M Ara-C. After four days of culture, the percentage of surviving cells in the cultures treated with anti-ITGA6 or anti-ITGB4 antibodies were significantly reduced in the two EVI1 and primary AML cell lines compared with the control cells (**Figs. 5A to D**). Because similar results were also obtained using the anti-cancer drug, VP-16 (**Fig. S4**), the treatment with anti-ITGA6 or anti-ITGB4 antibodies or anti-cancer drugs might recover the drug-sensitivity of EVI1^high^ leukemia cells.

To determine whether the effect of Ara-C was dependent on the cell adhesion ability, AML1/shITGB4, AML1/shEVI1 and control AML1/shLuc cells were cultured on matrigel-coated or control BSA-coated plates and treated with Ara-C for three days; we could not use AML1/shITGA6 cells in this experiment. After three days of culture, the number of viable AML1/shLuc cells was significantly higher when the cells were cultured on matrigel-coated plates compared with conventional culture; however, the percentage of surviving AML1/shITGB4 cells was reduced at each time point.(**Fig. 5E**). The survival rate of the AML1/shEVI1 cells in both the conventional and matrigel-coated cultures was reduced compared with those of the AML1/shLuc and AML1/shITGB4 cells upon Ara-C treatment, whereas there was no difference in the survival rates of the AML1/shEVI1 cells cultured under conventional and matrigel-coated methods under Ara-C treatment. These results suggest that the drug resistance of EVI1^high^ leukemia cells is highly dependent on EVI1 expression and partially dependent on ITGB4 expression.

### EVI1^high^ leukemia cells become more quiescent when cultured on the matrigel-coated plates compared with those cultured on the BSA-coated plates

**Figure 6:**
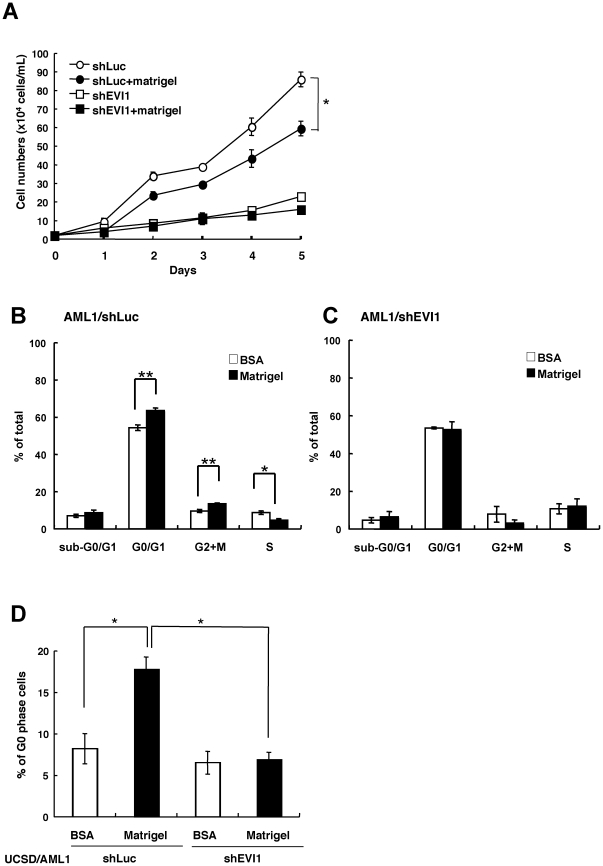
Decreased cell growth with increased cell population in G0-phase of EVI1^high^ AML cells cultured on the matrigel-coated plates. **A**. The UCSD/AML1 cells were transfected with shEVI1 (AML1/shEVI1) or control shLuc vector (AML1/shLuc) to determine their cell growth under normal culture conditions on BSA or matrigel-coated plates. **B and C**. The cell cycle of AML1/shLuc as a control (**B**) and AML1/shEVI1 (**C**) were analyzed using BD FACSCalibur after double staining with BrdU-APC and 7-AAD. The percentage of viable cells in each cell cycle is indicated with white (BSA-coated) and black (matrigel-coated) bars. **D**. The percentage of AML1/shEVI1 (black bars) and AML1/shLuc (white bars) cells in the G0 phase cultured on matrigel or BSA-coated plates were analyzed using BD FACSCalibur after double staining with Ki67-Alexa647 and 7-AAD. Each experiment was performed in triplicate, and the experiments were independently repeated at least three times. The results are shown as the mean ± S.E. The statistical analysis was performed using the Student's *t-test* (**p<0.01; **p<0.05, vs. control).

To confirm the advantages of cell adhesion in drug-responsiveness, we determined the growth and cell cycle status of AML cells with EVI1^high/low^, which were cultured on matrigel or BSA-coated plates. As shown in **Fig. 6A**, the UCSD/AML cells with EVI1^high^ expression (AML1/shLuc) on the matrigel-coated plates grew more faster rapidly on the matrigel-coated plates than that on the BSA-coated plates, although the growth rate of the AML1/shLuc cells was faster than that of the AML1/shEVI1 cells. The cell cycle analysis represented revealed that, relative to AML1/shLuc cells cultured on the BSA-coated plates, the population of EVI1^high^ AML1 cells in G0/G1 and G2/M phase increased on the matrigel-coated plates, while the S-phase populations were decreased; however, each cell cycle population of AML1/shEVI1 cells did not change under either culture condition (**Fig. 6B and C**). Moreover, the population of AML1/shLuc cells in the G0 phase was significantly increased on the matrigel-coated plates compared with the same cells on the BSA-coated plates; however, the population of AML1/shEVI1 cells in the G0 phase was the same under both culture conditions (**Fig. 6D**). Because the population of U937 cells with high EVI1 expression (U937/EVI1) in the G0-phase was increased on the matrigel-coated plates compared with those on the BSA-coated plates (**Fig. S5**), the increased quiescence of adhered EVI1^high^ AML cells might be one of the reasons for resistance to anti-cancer drugs.

## Discussion

Resistance to treatment with anti-cancer drugs is determined by a variety of factors, including somatic cell genetic differences in tumors and the phenotypes of leukemia stem cells (LSC). LSCs can infiltrate bone marrow niches, leading to enhanced self-renewal and proliferation, and enforced quiescence; the resistance to chemotherapeutic agents and adhesion molecules, such as VCAM-1 and VLA-4, has been described in the localization and retention of normal HSCs and/or leukemia cells within the bone marrow niche [19]. In this manuscript, we examined the cell adhesion ability of EVI1^high^ leukemia cells because the increased expression of EVI1 in AML is a well-known prognostic factor for poor outcome and is associated with drug resistance [4], [5]. We found that the EVI1^high^ leukemia cells with increased ITGA6 and ITGB4 expression exhibited stronger adhesion to laminin complexes than the EVI1^low^ leukemia cells; although, the EVI1^low^ leukemia cells exhibited increased adhesion to fibronectin. Moreover, the interaction between EVI1^high^ leukemia and MC3T3-E1 osteoblastic cells was partially dependent on laminin-332. The downregulation of EVI1 or ITGB4 in UCSD/AML1 leukemia cells restored chemo-sensitivity. The knockdown of ITGA6 expression in EVI1^high^ leukemia cells greatly reduced their survival in culture; thus, we conclude that the increased cell adhesion ability of EVI1^high^ leukemia cells is primarily dependent on the expression of ITGA6, and that signaling from IGTA6 is crucial for the survival of EVI1^high^ leukemia cells.

The expression of the ITGA6/ITGB4 complex could promote tumor progression and the metastasis of various cancer cells, including breast, colorectal, and thyroid carcinomas. Moreover, the laminin receptor for ITGA6 is ubiquitously expressed in human and mouse hematopoietic stem and progenitor cells [26]. In combination with ITGA4, ITGA6 expression in hematopoietic stem and progenitor cells is believed to create a homing receptor for short-term stem cells [27]. ITGA6 functions during the homing of fetal liver HPCs but not during the homing and engraftment of multilineage repopulating HSCs *in vivo* [28]. Therefore, ITGA6 might function as a laminin receptor to sustain HSCs in the bone marrow niche. We are now developing anti-human ITGA6/ITGB4 complex antibodies, which is a new therapy for refractory AML. In preliminary experiments, we demonstrated that the number of EVI1^high^ AML cells was significantly decreased in the bone marrow after the intravenous injection of anti-human ITGA6/ITGB4 complex antibodies into NOG immune deficient mice (data not shown). As a corollary, the increased expression of the ITGA6/ITGB4 complex in EVI1^high^ leukemia might be an important factor in maintaining leukemia stem cells in the bone marrow.

The adhesion of EVI1^high^ AML cells became more resistance to anti-cancer drug therapy, and the population of cells in the G0-phase was increased. Because the increased population of G0-phase cells is likely dependent on the expression of EVI1, future research should focus on the characterization of the mechanism underlying the increased quiescent of cells through EVI1 expression subsequent to their adhesion to matrigel. Moreover, the induction of PI-3K/AKT/Bcl-2 signaling through the VLA-4-fibronectin interaction results in resistance to anoikis and drug-induced apoptosis [21]; we also showed the cell adhesion of EVI1^high^ AML cells to matrigel induced expression of BCL and phosphorylation of AKT (data not shown). Therefore, the adhesion of AML cells may provide advantages to the surviving cells, and the mechanism of drug-resistance is potentially much more complicated; however, we should characterize the survival mechanism of leukemia cells in the bone marrow niche.

To support our data, we examined the expression patterns of ITGA6/ITGB4 and VLA-4 (ITGA4/ITGB1) heterodimers by comparing the gene expression profiles of EVI1^high^ and EVI1^low^ AML deposited in Gene Expression Omnibus (GSE6891, GEO in NCBI website) [29]. As shown in **Fig. S6**, the expression of ITGA6 was significantly higher in the 12 cases of EVI1^high^ AML than in the 10 cases of EVI1^low^ AML (p<0.05); however, the differences in expression of ITGB4, ITGA4 and ITGB1 did not account for the differences between the two groups. We also determined the expression patterns of four integrin genes from the expression profiles of AML cases in remission and relapse that were deposited in GEO at NCBI (GDS1059) [30]. Based on the expression profiles, the expression of ITGA6 was significantly higher in the 28 cases of AML with relapse than in the 25 cases with remission (p<0.05); however, the differences in expression of ITGB4, ITGA4 and ITGB1 did not account for the difference between the two groups. These results show that the increased expression of ITGA6 is an important marker in both EVI1^high^ and relapsed AML, which suggests that ITGA6 might be an important target for the molecular-targeted treatment of refractory leukemia, including EVI1^high^ AML.

## Materials and Methods

### Cell lines and culture conditions

The MOLM1, HNT34, U937, K562, and HL60 cells were cultured in RPMI1640 medium supplemented with 10% fetal calf serum at 37°C and 5% CO_2_. The UCSD/AML1, PT9 and PT11 cells [24] were cultured in RPMI1640 medium supplemented with 10% fetal calf serum and human granulocyte-macrophage colony stimulating factor (GM-CSF, 1 ng/ml) at 37°C and 5% CO_2_. The MOLM1 cells were purchased from the Hayashibara Institute, and the MC3T3-E1 and HNT34 cells were purchased from the RIKEN cell bank. The 293T cells were cultured in high-glucose DMEM supplemented with 10% fetal calf serum, and the MC3T3-E1 cells were cultured in MEM alpha supplemented with 10% fetal calf serum.

### Oligonucleotide microarray

This experimental method was previously described [24].

### Adhesion-mediated drug resistance (CAM-DR) in AML cell lines

The UCSD/AML1, MOLM1, PT9 and PT11 cells were plated onto tissue culture plates with BSA or matrigel-coated wells and subsequently incubated with 10^−3^ to 10^−7^ M cytosine-arabinoside (Ara-C) for 48 h. The number of viable cells was determined using trypan blue exclusion.

### Neutralizing Antibody

The neutralizing antibodies used included rat anti-ITGA6 (Santa Cruz) and mouse anti-ITGB2, ITGB3 and ITGB4 (Millipore).

### PCR and PCR primers

Total cellular RNA was isolated using Trizol (Invitrogen), and the cDNA was prepared using oligo(dT) primer and reverse transcriptase (SuperScript; Invitrogen). The polymerase chain reaction (PCR) was performed using Ex Taq (Takara-Bio) on a GeneAmp 2400 machine (Applied Biosystems). The primers for all integrin family members, EVI1 and b-actin were produced based on methods used in previous studies [12], [31]. The primers for N and VE-cadherin were produced based on the Quantitative PCR Primer Database (http://lpgws.nci.nih.gov/cgi-bin/PrimerViewer). The primers for the laminin family genes were produced based on Primer Bank (http://pga.mgh.harvard.edu/primerbank/).

### Introduction of small hairpin RNAs for EVI1, ITGA6, ITGB1 and ITGB4 in UCSD/AML1 cells

The pSIREN-retroQ-ZsGreen plasmid (Takara-Bio) was used to inhibit EVI1 expression, and expression of ZsGreen (green fluorescent protein) was used as a marker. The following sequences were cloned into the BamHI-EcoRI sites of the pSIREN-retroQ-ZsGreen plasmid to create shRNAs against human EVI1 [32], 5′-GATCGCTCTAAGGCTGAACTAGCAGTTCAAGAGACTGCTAGTTCAGCCTTAGATTTTTTG-3′; human ITGA6, 5′-GATCTCCTTTCAGGTTCAGTAGTTATTTCAAGAGAATAGTTACTGAATCTGAGAGGTTTTTG-3′; human ITGB1, 5′-GATCTGGAGGGTTGTTTCGGGTTTCATTCAAGAGATGAAGTCCGAAGTAATCCTCCTTTTTG -3′; and human ITGB4, 5′-GATCGCCAGCGACTACACTATTGGATCTCGAGATCCAATAGTGTAGTCGCTGGTTTTTG (MISSION shRNA library, Sigma). We used the pSIREN-retroQ-ZsGreen-shLuc plasmid containing an shRNA against firefly luciferase (Takara-Bio) as a control. The retroviral particles were generated using the p10A1 packaging vector (Takara-Bio) via the transient transfection of 293T cells. The transfection was conducted using Hilymax liposome transfection reagent (Dojin). For retroviral transduction, 1×10^6^ cells were plated onto a 6-cm dish containing 5 ml retroviral supernatant containing 100 ng/ml of polybrene for 24 h. After two weeks, the ZsGreen-positive cells were sorted using a JSAN cell sorter (Bay Bioscience). The reduction of EVI1 or ITGB4 expression was confirmed using RT-PCR.

### Establishment of stable U937 cell lines expressing EVI1

The pGCDNsam-EVI1-IRES-EGFP construct was kindly provided by Dr. A. Iwama (Chiba University, Chiba, Japan). To produce recombinant retrovirus, the plasmid DNA was transfected into 293GP cells along with the vesicular stomatitis virus G (VSV-G) expression plasmid via CaPO_4_ precipitation. For retroviral transduction, 1×10^5^ U937 cells were plated onto 96-well flat-bottomed plates and infected for 24 h with either an EVI1 retroviral supernatant (pGCDNsam-EVI1-IRES-EGFP) or a control retroviral supernatant (pGCDNsam-IRES-EGFP) containing 100 ng/ml of polybrene. After 7 days, the green fluorescent protein (GFP)-positive U937 cells were sorted using a JSAN cell sorter (Bay Bioscience, Kobe, Japan).

### Laminin-332 (laminin-5) knockdown in MC3T3-E1 cells

The pSIREN-retroQ-ZsGreen plasmid (Takara-Bio) was used to inhibit the expression of the a3 chain of laminin-332, and the expression of ZsGreen (GFP) was used as a marker. The following sequence was cloned into the BamHI-EcoRI sites of the plasmid to create an shRNA against the a3 chain of murine laminin-332: 5′-GATCTGAACTCCTTAAATGATTATGAATTCAAGAGATTCATAATCATTTAAGGAGTCTTTTTTACGCGTG-3′. The target sequence for the knockdown was determined using the BLOCK-iT RNAi Designer (http://www.invitrogen.com/rnai). We used pSIREN-retroQ-ZsGreen-shLuc containing shRNA against firefly luciferase (Takara-Bio) as a control. We transiently introduced the laminin-332 or luciferase shRNA expression vector into MC3T3-E1 cells using an Amaxa Nucleofector (Lonza) and determined the expression levels of the a3 and g2 subunits of laminin-332.

### Cell proliferation and cell viability assays

A cell proliferation assay was performed using a Cell Counting Kit-8 (Dojin) according to the manufacturer's protocol. Briefly, 5×10^3^ cells/well were plated onto a 96-well culture plate and incubated for 1–3 days. Approximately 10 ml of Cell Counting Kit-8 reagent was added to the plate, and the absorbance at 450 and 620 nm was measured using an Immunomini NJ2300 plate reader (Nunc). A cell viability assay was performed using the Cell Counting Kit-8 reagent according to the manufacturer's protocol. For this assay, 5×10^3^ cells were plated onto a 96-well culture plate containing Ara-C (1×10^−6^ M), rat anti-ITGA6 (20 mg/ml) or mouse anti-ITGB4 (20 mg/ml). The cells were incubated for 1–2 days and 10 ml of Cell Counting kit-8 reagent was added. The absorbance at 450 and 620 nm was measured. The cell viability was calculated by dividing the value obtained for the treatment well by the average value obtained for the control wells. For cell viability assay, cells were seeded in 5×10^4^ cells/ml to matrigel coated culture plate. Cells were co-cultured with Ara-C (1×10^−6^ M) and anti-ITGA6 antibody (20 mg/ml) or anti-ITGB4 (20 mg/ml) or isotype control IgG (20 mg/ml). After incubation for 1 to 4 days, living cell numbers were counted by trypan blue exclusion. The cell viability was calculated by dividing the value for the treatment well by the value for the control wells. Each experiment was performed in triplicate. Data are shown as mean ± S.E. Statistical analysis was performed using Student's *t-test*.

### Cell adhesion assay

Four types of extracellular matrices (ECM; matrigel, fibronectin, laminin, collagen) and BSA (as a control) were used for the cell adhesion assay. A culture plate was coated overnight with a 1/40 dilution of Growth Factor Reduced-Matrigel (BD Falcon), 5 mg/cm^2^ of fibronectin (BD Falcon), 5 mg/cm^2^ of laminin (BD Falcon), 0.3 mg/ml of collagen (Nitta Gelatin) or 5% w/v BSA (Nacalai Tesque). After coating with ECM, 1×10^5^ cells/ml were transferred to each pre-coated well and incubated for 24 h at 37°C. After washing two times with phosphate buffered saline (PBS), the floating and adherent cells were counted using trypan blue, and the number of adherent cells was divided by the number of floating cells to calculate the cell adherence ratios. In the neutralization experiments, the cells were pre-incubated with 20 mg/ml of anti-ITGA6, ITGB2, ITGB3 or ITGB4 antibodies prior to plating on the matrigel [33].

### FACS analysis

The intensity of ITGB3, ITGB4 and ITGA6 expression in UCSD/AML1 and U937 cells was analyzed using FACSCalibur (BD Bioscience). The cells were stained with phycoerythrin (PE)-labeled anti-human ITGA6 (Clone: GoH3), ITGB4 (Clone: 58XB4) or ITGB3 (BI-PL2) antibodies purchased from Biolegend, Inc.

### BrdU (Bromodeoxyuridine)/7-AAD (7-Aminoactinomycin D) staining

To assess the percentage of cells in sub G0/G1, G0/G1, and G2/M and S phase after 48 h of culture on matrigel or BSA-coated plates, 1×10^6^ cells from each culture were fixed in BD Cytofix/Cytoperm buffer for 20 min., followed by a 300-mg/ml DNase treatment for 30 min. The cells were subsequently washed twice in BD Perm/Wash Buffer and stained with anti BrdU-APC (BD Biosciences/PharMingen, San Jose, CA) for 15 minutes, followed by staining with 7-AAD (BD Biosciences/PharMingen, San Jose, CA). The cells were harvested and analyzed using FACSCalibur with FL-4 (for BrdU-APC) and FL-3 (for 7-AAD) settings.

### Ki67/7AAD staining

To assess the percentage of cells in G0 phase after 48 h of culture on matrigel or BSA-coated plates, 1×10^6^ cells from each culture were fixed in 70% ethanol for 2 h, followed by a 2.5 µg/ml RNAse treatment for 30 minutes. The cells were subsequently washed twice in PBS/1% fetal bovine serum (FBS) and stained with mouse anti-human Ki67 (BD Biosciences/PharMingen, San Jose, CA) and Alexa647-conjugated anti-mouse IgG (Invitrogen) for 30 minutes, followed by staining with 7-AAD (BD Biosciences/PharMingen, San Jose, CA). The cells were harvested and analyzed using FACSCalibur with FL-4 (for Ki67-Alexa647) and FL-3 (for 7-AAD) settings.

### Statistical analysis

Student's t-test was used to compare differences between the two groups.

## Supporting Information

Figure S1 **Expression of integrin genes and cell binding ability of various myeloid leukemia cells.** **A**. The expression of 25 members of the integrin family was detected using semi-quantitative RT-PCR in HL60, U937, and K562 (cell lines with EVI1^low^ expression) and in UCSD/AML1, MOLM1, and HNT34 (cell lines with EVI1^high^ expression). The expression of EVI1 and b-actin in these cells is also shown. **B**. The expression of 18 genes in the integrin family was detected using RT-PCR in AML1/shLuc and AML1/shEVI1 cell lines. **C and D**. The adhesion ability of various HNT-34 cell lines (Pt, parental;/shLuc, transfection with small hairpin RNA for firefly luciferase;/shEVI1, transfection with small hairpin RNA for EVI1) to bind to matrigel is shown in C, and the expression of integrin genes in conjunction with EVI1 and b-actin is shown in D. **E and F**. The ability of PT9-related (PT9/shLuc and PT9/shEVI1) and PT11-related (PT11/shLuc and PT11/shEVI1) cell lines to bind to matrigel was measured (E), and the expression of various integrin genes in these four cell lines was determined using RT-PCR (F) **G**. The expression patterns of integrin genes were determined in U937/GFP and U937/EVI1 cell lines in conjunction with b-actin and EVI1.
(DOC)

Figure S2 **Expression of various laminin chains in MC3T3-E1 cells.** The relative expression levels of laminin a chains 1 to 5, laminin b chains 1 to 3, and laminin g chains 1 to 3 in MC3T3-E1 cells were determined using semi-quantitative RT-PCR.
(DOC)

Figure S3 **Dose-response functions for AraC against four EVI1high AML cells.** UCSD/AML1, MOLM1, PT9 and PT11 cells were incubated in BSA- or matrigel-coated wells of tissue culture plates and subsequently incubated with 10-3 to 10-7 M cytosine-arabinoside (Ara-C) for 48 h. Relative cell viability is calculated as a percentage relative to standard controls. Data are shown as mean ± S.E. Statistical analysis was performed using Student's t-test. A star (*) indicates p<0.05 and double stars (**) indicate p<0.01.
(DOC)

Figure S4 **Drug sensitivity of EVI1^high^ and EVI1^low^ leukemia cells cultured with or without MC3T3-E1 cells determined by treatment with VP-16 or Ara-C.** UCSD/AML1 (EVI1^high^) leukemia cells and HL60 (EVI1^low^) leukemia cells were treated with Ara-C or VP-16 for three days under plastic flasks (open diamonds) or co-cultured with MC3T3-E1 cells (closed squares); the viable cells were counted at each indicated time point. The percent cell viability compared to the number of untreated cells is shown for each indicated day. A star (*) indicates p<0.05.
(DOC)

Figure S5 **Decreased cell growth with increased cell population in G0-phase of U937 cells with EVI1 expression cultured on the matrigel-coated plates.** A. U937 cells were introduced by EVI1 expression vector (U937/EVI1) or control vector (U937/GFP) to determine their cell growths with culture condition on BSA or matrigel-coated plates. B and C. Cell cycle of U937/GFP as a control (B) and U937/EVI1 (C) were analyzed by BD FACSCalibur after double-stained by BrdU-APC and 7-AAD. Percentages of each cell cycles were shown by white bars (BSA-coated) and black bars (matrigel-coated). D. The percentage of cells in G0 phase in U937/EVI1 (black bars) and U937/GFP cells (white bars) cultured on matrigel- or BSA-coated plates were analyzed by BD FACSCalibur after double-stained by Ki67-Alexa647 and 7-AAD. Each experiment was performed in triplicate, and experiments were independently repeated at least three times. Results are shown as mean ± S.E. Statistical analysis was performed using Student's t-test (**p<0.01; **p<0.05, vs control).
(DOC)

Figure S6 **Expression profiles of ITGA6 and ITGB4 in AML patients.** A and B. The expression patterns of ITGA6 (A) and ITGB4 (B) are shown as gene expression profiles for ten AML patients with EVI1low and ten AML patients with EVI1high expression (http://www.ncbi.nlm.nih.gov/geo, accession number GSE6891 [NCBI GEO]). C and D. The expression patterns of ITGA6 (C) and ITGB4 (D) are shown as gene expression profiles for patients in remission and for AML patients who had relapsed (http://www.ncbi.nlm.nih.gov/geo, accession number GDS1059 [NCBI GEO]).
(DOC)
