# Supplementary figures and images for: The Increased Expression of Integrin α6 (ITGA6) Enhances Drug Resistance in EVI1high Leukemia
Source: PLoS One. 2012 Jan 25;7(1):e30706. doi: 10.1371/journal.pone.0030706 (PMC3266272; doi:10.1371/journal.pone.0030706)

**Figure S1.**


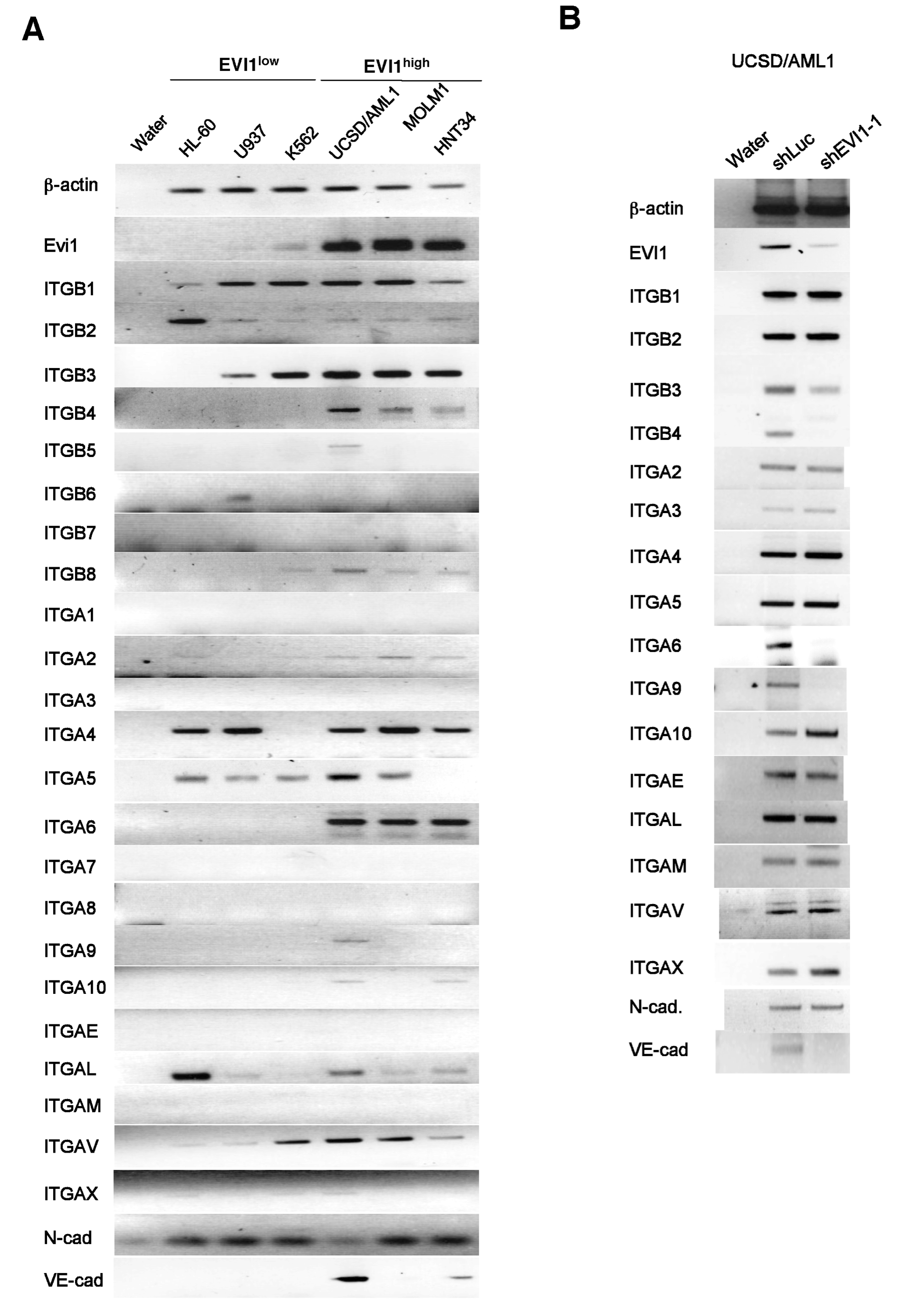


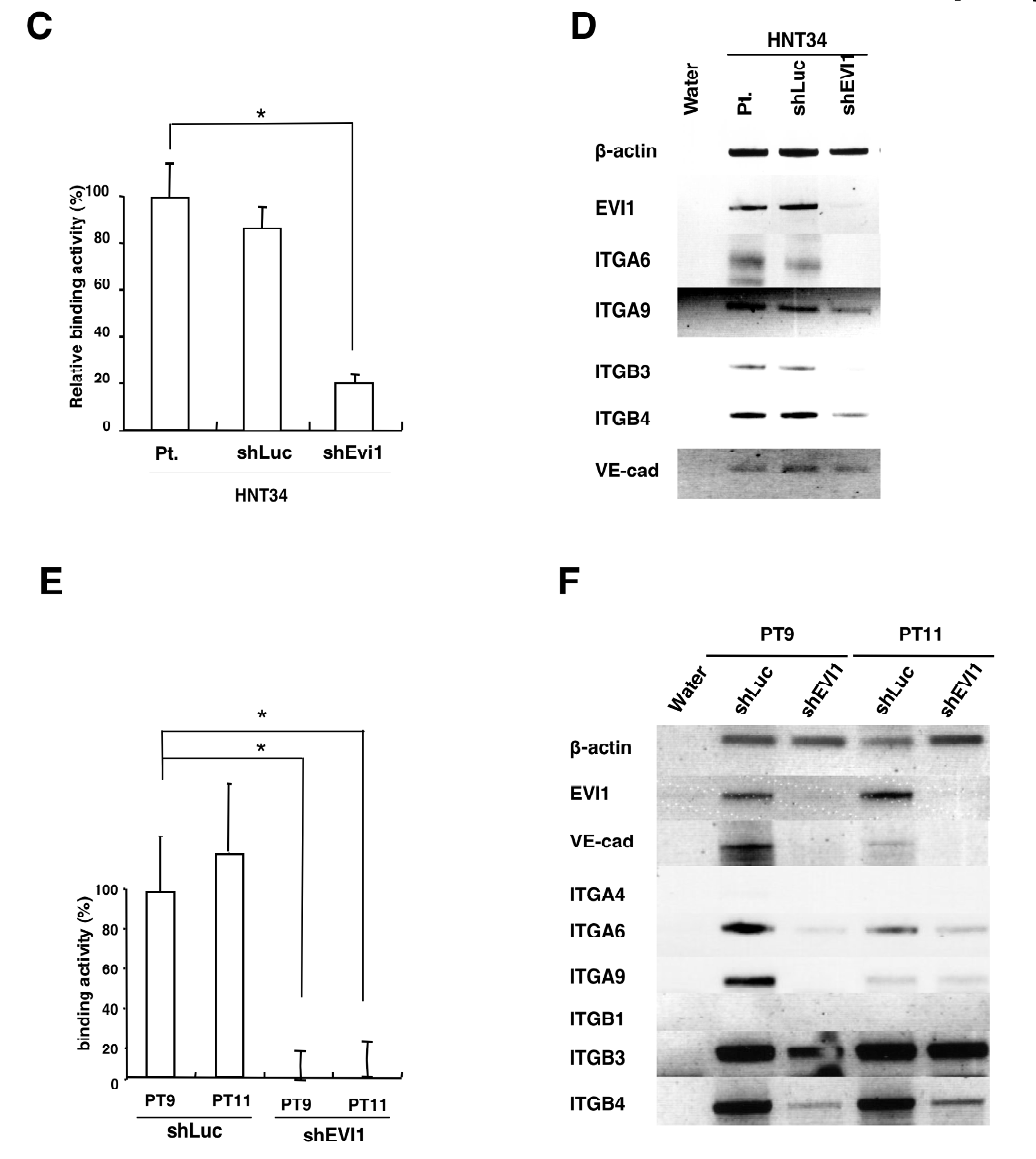


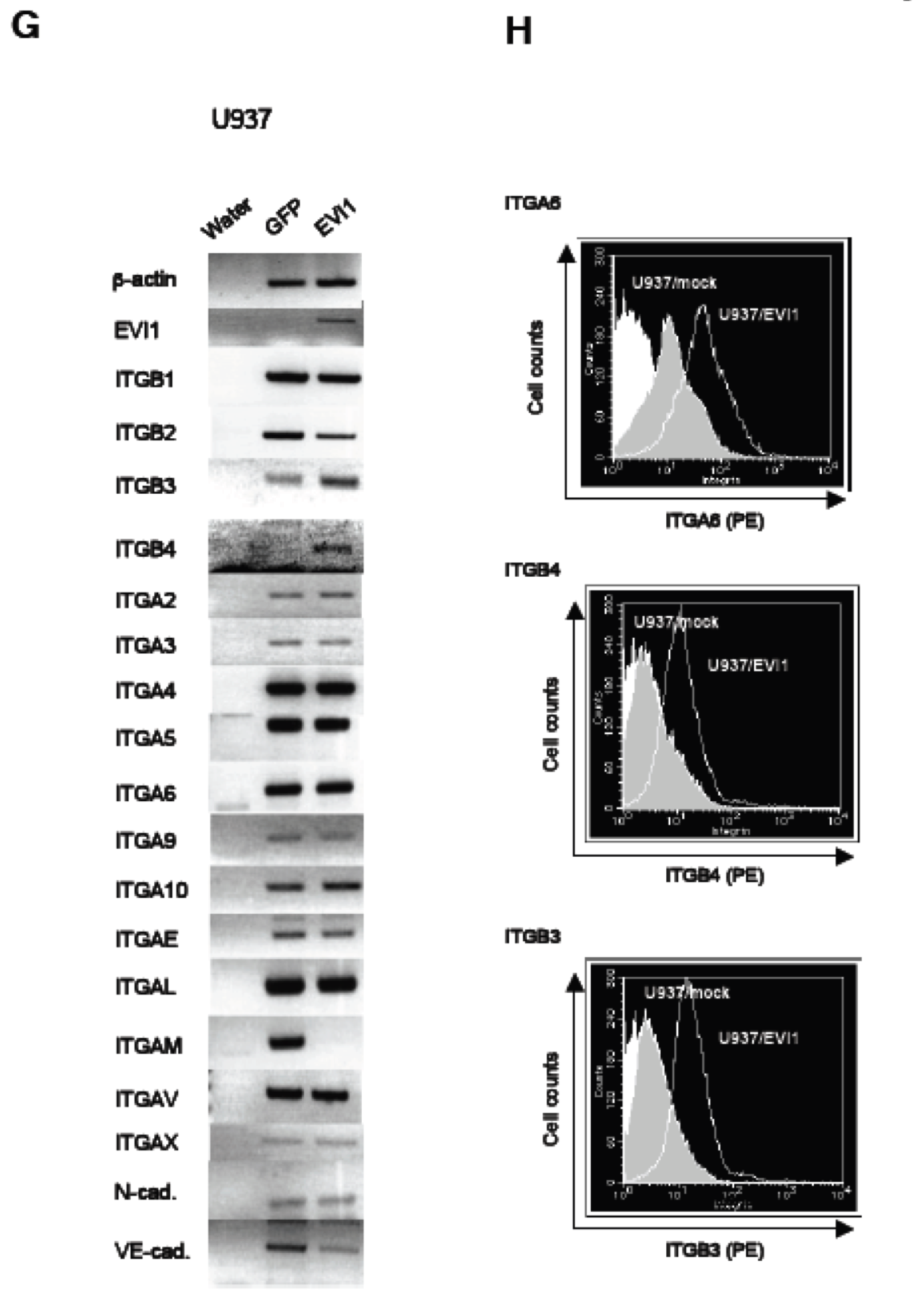

Supplement: Figure S1 — Expression of integrin genes and cell binding ability of various myeloid leukemia cells. A. The expression of 25 members of the integrin family was detected using semi-quantitative RT-PCR in HL60, U937, and K562 (cell lines with EVI1low expression) and in UCSD/AML1, MOLM1, and HNT34 (cell lines with EVI1high expression). The expression of EVI1 and b-actin in these cells is also shown. B. The expression of 18 genes in the integrin family was detected using RT-PCR in AML1/shLuc and AML1/shEVI1 cell lines. C and D. The adhesion ability of various HNT-34 cell lines (Pt, parental;/shLuc, transfection with small hairpin RNA for firefly luciferase;/shEVI1, transfection with small hairpin RNA for EVI1) to bind to matrigel is shown in C, and the expression of integrin genes in conjunction with EVI1 and b-actin is shown in D. E and F. The ability of PT9-related (PT9/shLuc and PT9/shEVI1) and PT11-related (PT11/shLuc and PT11/shEVI1) cell lines to bind to matrigel was measured (E), and the expression of various integrin genes in these four cell lines was determined using RT-PCR (F) G. The expression patterns of integrin genes were determined in U937/GFP and U937/EVI1 cell lines in conjunction with b-actin and EVI1. (DOC) [file pone.0030706.s001.doc]

**Figure S2.**


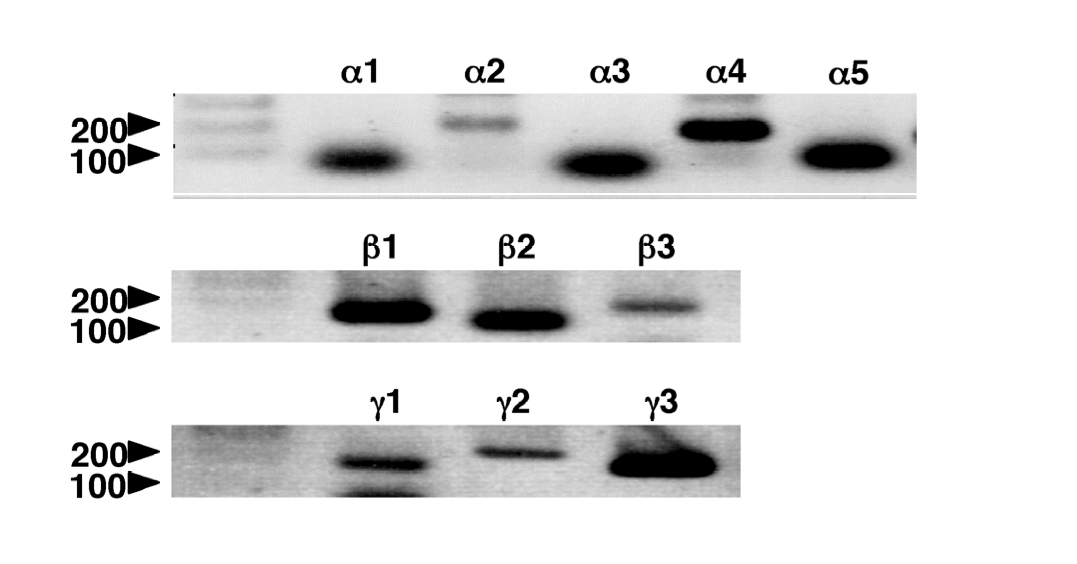

Supplement: Figure S2 — Expression of various laminin chains in MC3T3-E1 cells. The relative expression levels of laminin a chains 1 to 5, laminin b chains 1 to 3, and laminin g chains 1 to 3 in MC3T3-E1 cells were determined using semi-quantitative RT-PCR. (DOC) [file pone.0030706.s002.doc]

**Figure S3.**


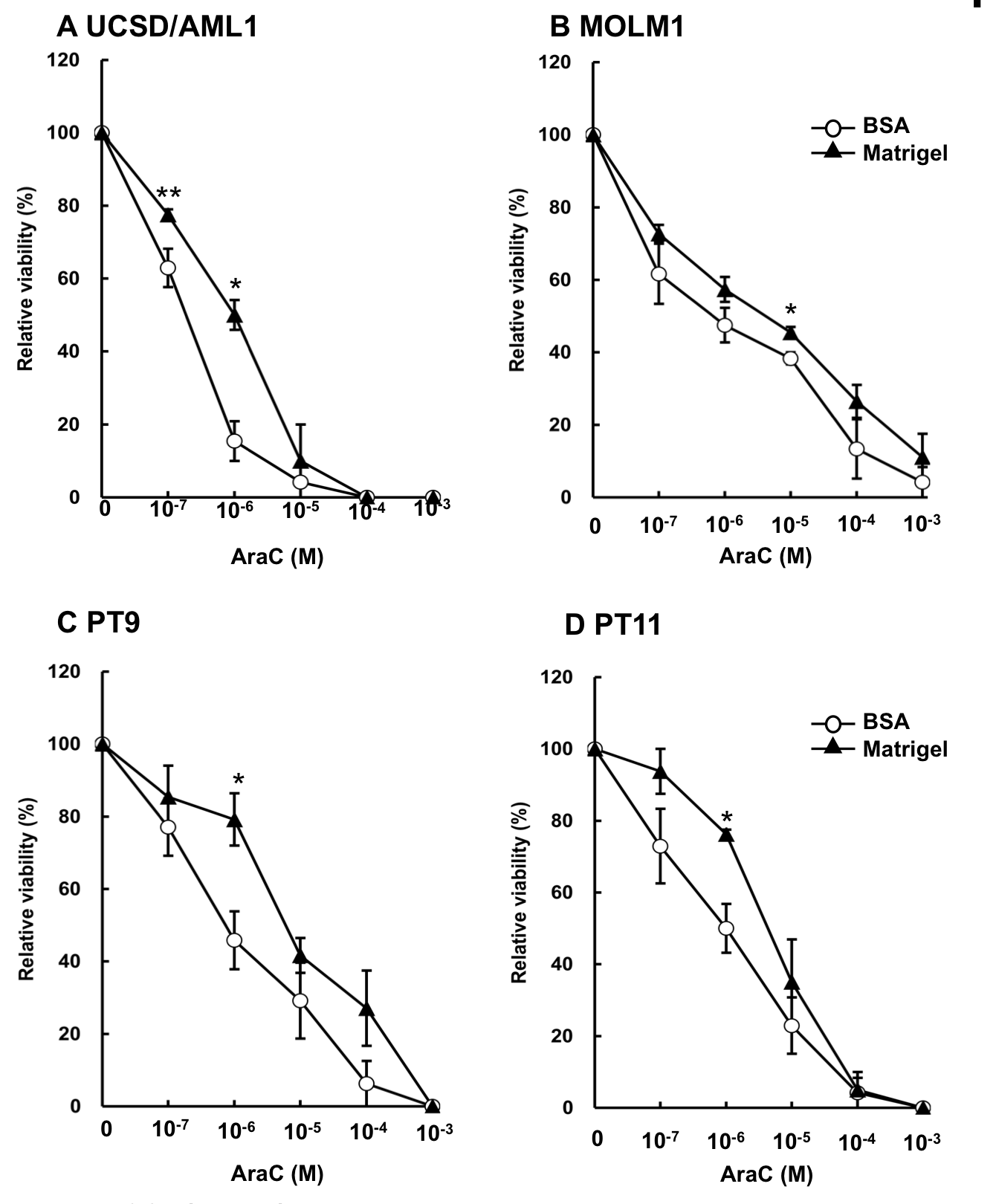

Supplement: Figure S3 — Dose-response functions for AraC against four EVI1high AML cells. UCSD/AML1, MOLM1, PT9 and PT11 cells were incubated in BSA- or matrigel-coated wells of tissue culture plates and subsequently incubated with 10-3 to 10-7 M cytosine-arabinoside (Ara-C) for 48 h. Relative cell viability is calculated as a percentage relative to standard controls. Data are shown as mean ± S.E. Statistical analysis was performed using Student's t-test. A star (*) indicates p<0.05 and double stars (**) indicate p<0.01. (DOC) [file pone.0030706.s003.doc]

**Figure S4.**


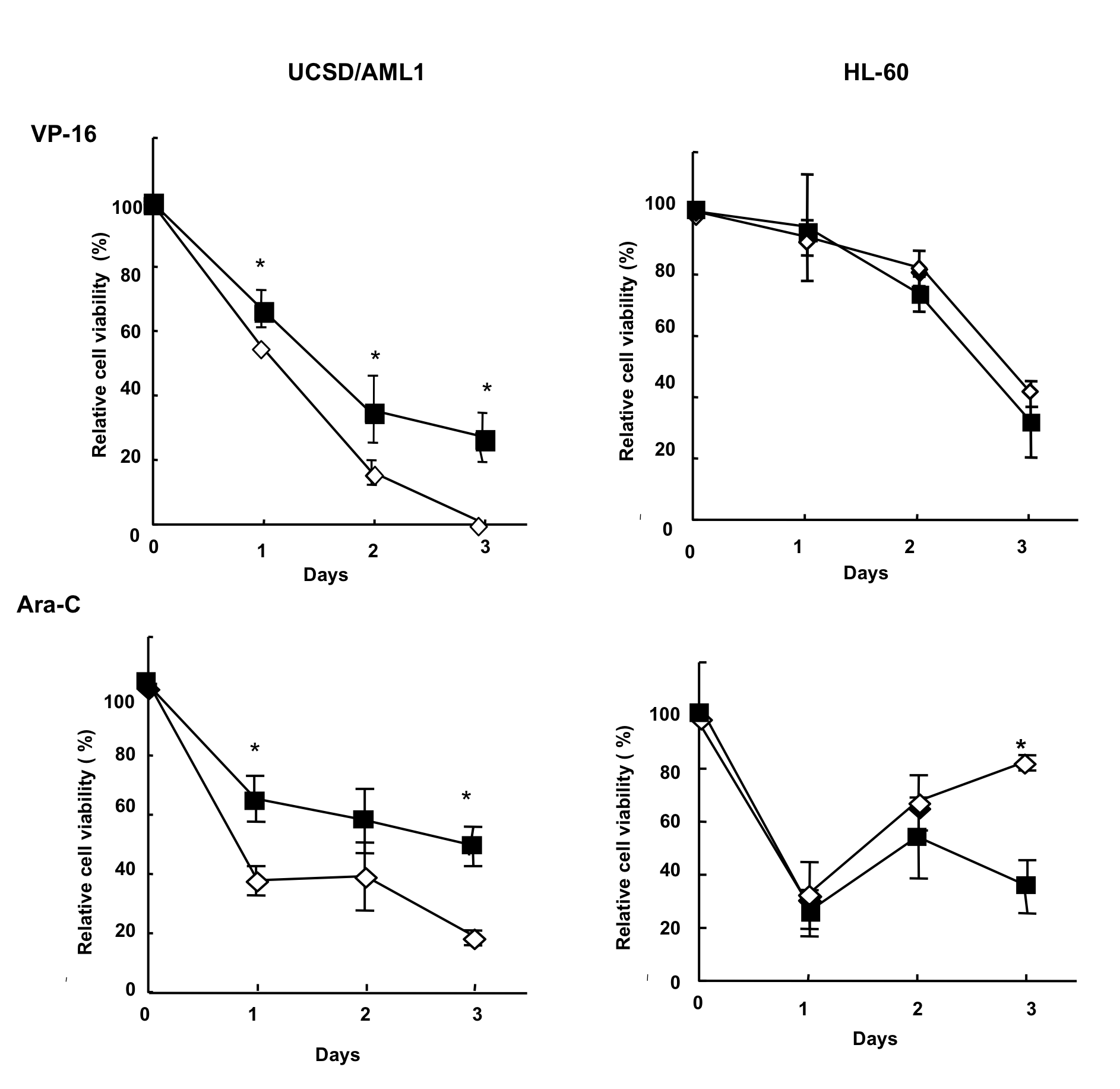

Supplement: Figure S4 — Drug sensitivity of EVI1high and EVI1low leukemia cells cultured with or without MC3T3-E1 cells determined by treatment with VP-16 or Ara-C. UCSD/AML1 (EVI1high) leukemia cells and HL60 (EVI1low) leukemia cells were treated with Ara-C or VP-16 for three days under plastic flasks (open diamonds) or co-cultured with MC3T3-E1 cells (closed squares); the viable cells were counted at each indicated time point. The percent cell viability compared to the number of untreated cells is shown for each indicated day. A star (*) indicates p<0.05. (DOC) [file pone.0030706.s004.doc]

**Figure S5.**

**
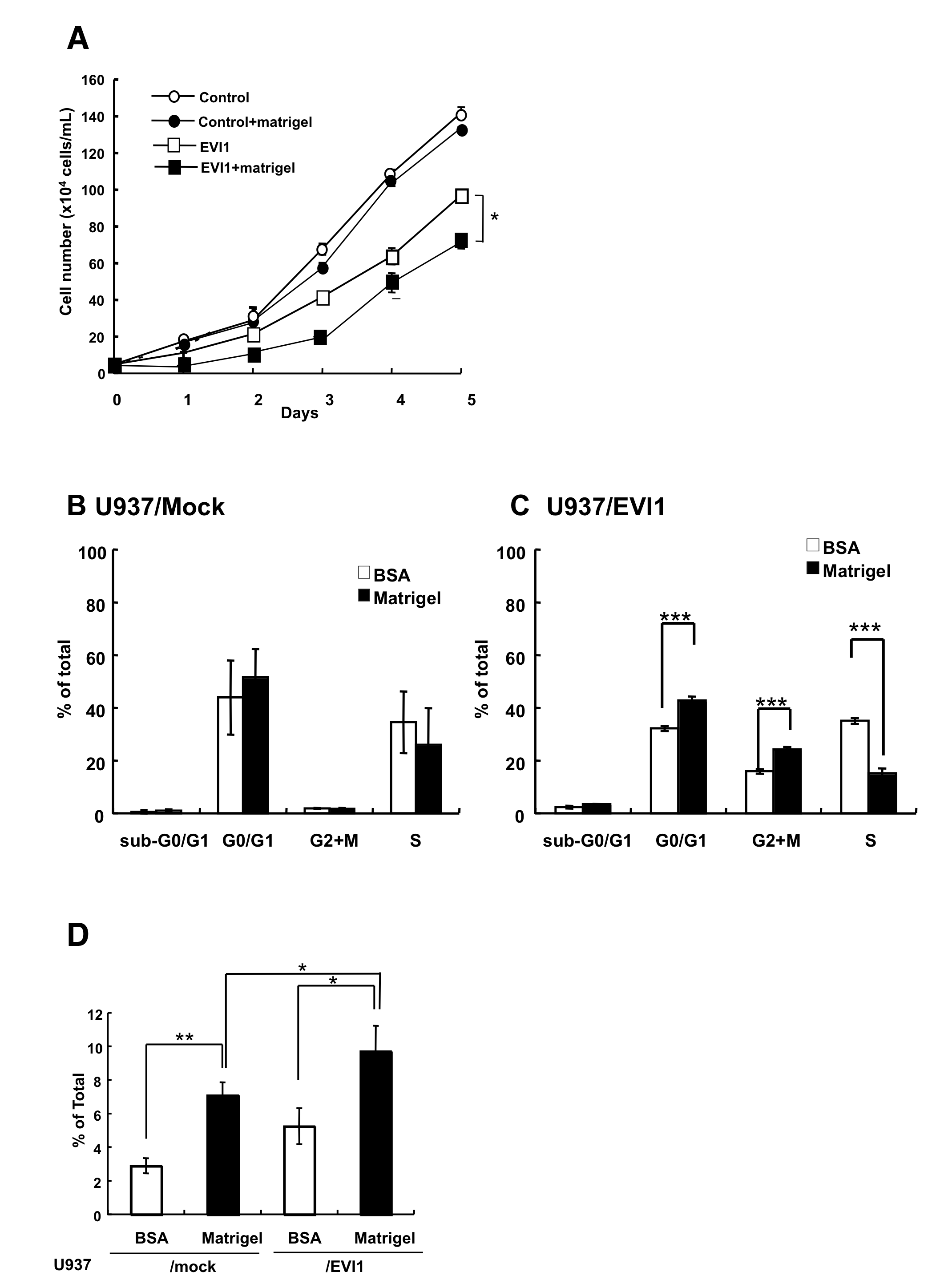
**

Supplement: Figure S5 — Decreased cell growth with increased cell population in G0-phase of U937 cells with EVI1 expression cultured on the matrigel-coated plates. A. U937 cells were introduced by EVI1 expression vector (U937/EVI1) or control vector (U937/GFP) to determine their cell growths with culture condition on BSA or matrigel-coated plates. B and C. Cell cycle of U937/GFP as a control (B) and U937/EVI1 (C) were analyzed by BD FACSCalibur after double-stained by BrdU-APC and 7-AAD. Percentages of each cell cycles were shown by white bars (BSA-coated) and black bars (matrigel-coated). D. The percentage of cells in G0 phase in U937/EVI1 (black bars) and U937/GFP cells (white bars) cultured on matrigel- or BSA-coated plates were analyzed by BD FACSCalibur after double-stained by Ki67-Alexa647 and 7-AAD. Each experiment was performed in triplicate, and experiments were independently repeated at least three times. Results are shown as mean ± S.E. Statistical analysis was performed using Student's t-test (**p<0.01; **p<0.05, vs control). (DOC) [file pone.0030706.s005.doc]

**Figure S6.**


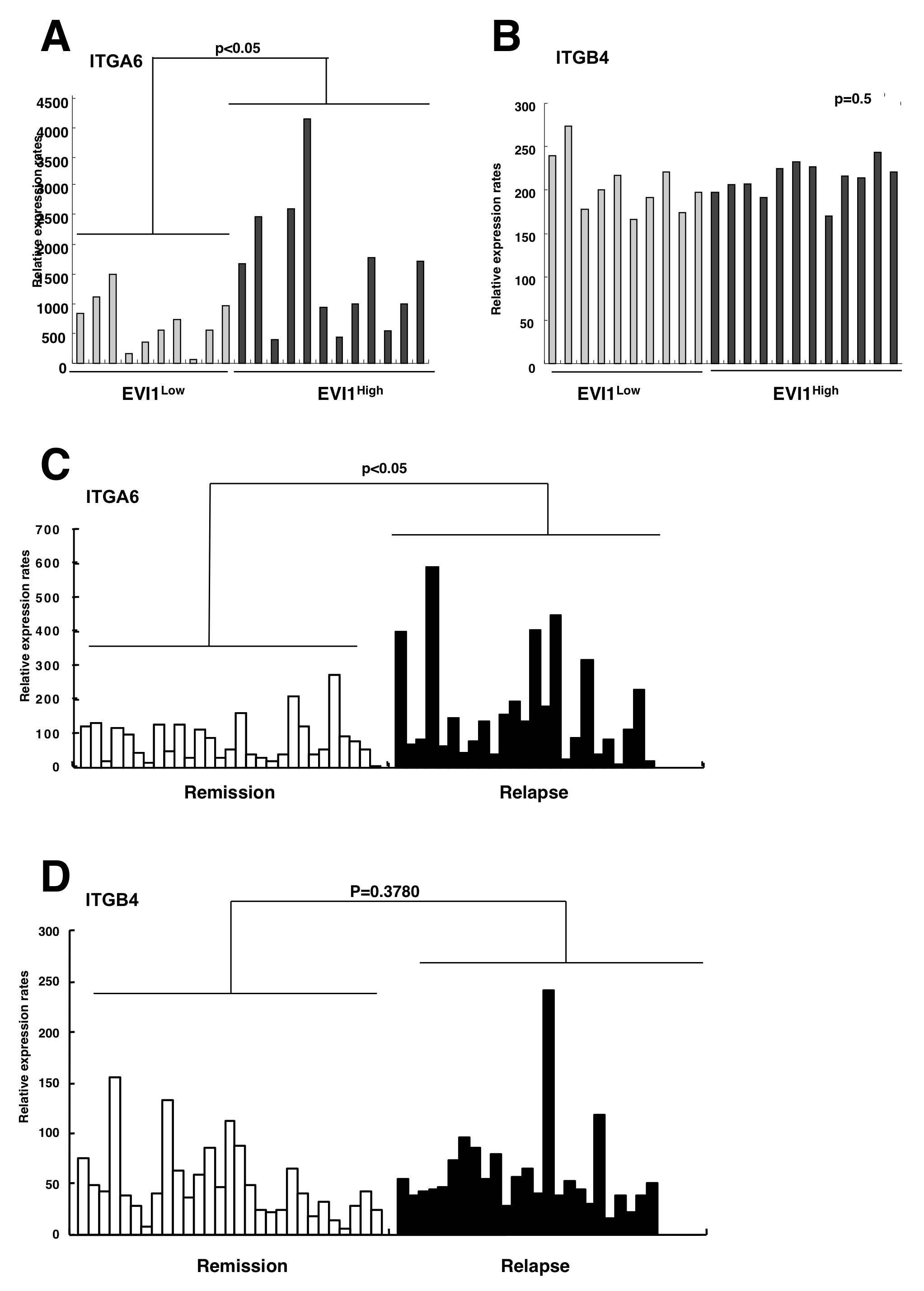

Supplement: Figure S6 — Expression profiles of ITGA6 and ITGB4 in AML patients. A and B. The expression patterns of ITGA6 (A) and ITGB4 (B) are shown as gene expression profiles for ten AML patients with EVI1low and ten AML patients with EVI1high expression (http://www.ncbi.nlm.nih.gov/geo, accession number GSE6891 [NCBI GEO]). C and D. The expression patterns of ITGA6 (C) and ITGB4 (D) are shown as gene expression profiles for patients in remission and for AML patients who had relapsed (http://www.ncbi.nlm.nih.gov/geo, accession number GDS1059 [NCBI GEO]). (DOC) [file pone.0030706.s006.doc]
